# Evidence-based discussion increases childhood vaccination uptake: a randomised cluster controlled trial of knowledge translation in Pakistan

**DOI:** 10.1186/1472-698X-9-S1-S8

**Published:** 2009-10-14

**Authors:** Neil Andersson, Anne Cockcroft, Noor M Ansari, Khalid Omer, Manzoor Baloch, Ari Ho Foster, Bev Shea, George A Wells, José Legorreta Soberanis

**Affiliations:** 1 Centro de Investigación de Enfermedades Tropicales (CIET), Universidad Autónoma de Guerrero, Calle Pino, El Roble, Acapulco, CP39640, Mexico; 2 CIETcanada, University of Ottawa, Stewart Street, Ottawa K1N 6Z5, Canada; 3 CIET in Pakistan, Gulshan-e-Iqbal, Karachi, Pakistan; 4 Ottawa Heart Institute, Ruskin Street, Ottawa, Canada

## Abstract

**Background:**

Childhood vaccination rates are low in Lasbela, one of the poorest districts in Pakistan's Balochistan province. This randomised cluster controlled trial tested the effect on uptake of informed discussion of vaccination costs and benefits, without relying on improved health services.

**Methods:**

Following a baseline survey of randomly selected representative census enumeration areas, a computer generated random number sequence assigned 18 intervention and 14 control clusters. The intervention comprised three structured discussions separately with male and female groups in each cluster. The first discussion shared findings about vaccine uptake from the baseline study; the second focussed on the costs and benefits of childhood vaccination; the third focussed on local action plans. Field teams encouraged the group participants to spread the dialogue to households in their communities. Both intervention and control clusters received a district-wide health promotion programme emphasizing household hygiene. Interviewers in the household surveys were blind of intervention status of different clusters. A follow-up survey after one year measured impact of the intervention on uptake of measles and full DPT vaccinations of children aged 12-23 months, as reported by the mother or caregiver.

**Results:**

In the follow-up survey, measles and DPT vaccination uptake among children aged 12-23 months (536 in intervention clusters, 422 in control clusters) was significantly higher in intervention than in control clusters, where uptake fell over the intervention period. Adjusting for baseline differences between intervention and control clusters with generalized estimating equations, the intervention doubled the odds of measles vaccination in the intervention communities (OR 2.20, 95% CI 1.24-3.88). It trebled the odds of full DPT vaccination (OR 3.36, 95% CI 2.03-5.56).

**Conclusion:**

The relatively low cost knowledge translation intervention significantly increased vaccine uptake, without relying on improved services, in a poor district with limited access to services. This could have wide relevance in increasing coverage in developing countries.

**Trial registration:**

ISRCTN12421731.

## Background

Childhood vaccination coverage is stagnating and even deteriorating in parts of Africa and South Asia [1]. In Pakistan's Balochistan province, for example, measles vaccination uptake fell from 70% in 2005-6 to 54% in 2006-7; full DPT vaccination uptake fell from 70% to 58% over the same period [2].

The reasons for the declining vaccination rates are uncertain but could include increasing transport costs and possibly decreasing encouragement from government health services to take up vaccination, sometimes even with cutbacks in service provision.

Most initiatives to increase coverage of childhood vaccination in developing countries involve improvement or extension of health services [3,4]. The short-term reality is that the service offer cannot improve without sizeable investment; health services are stretched to maintain their existing service offer, including childhood vaccination. Yet many households do not take up the existing offer of vaccination.

In practice, parents weigh up the cost of vaccinating their children now, against the discounted costs of a possible illness in the future [5-7]. Where household resources are scarce and little public attention is paid to vaccine preventable diseases, the present costs of vaccinating easily eclipse the discounted costs of the possible future disease [8,9]. As a consequence, many children do not receive vaccination.

We could identify only a handful of credible studies reporting knowledge translation interventions that increase vaccination uptake without requiring improvement of the service offer [10].

If non-vaccinating households could access and discuss accurate local information about actual costs and benefits, we hypothesised they might arrive at a cost-benefit equation more favourable to childhood vaccination. This should increase vaccination uptake compared with non-discussion communities even without increased access to services.

A randomised controlled trial tested our hypothesis in Lasbela, one of the poorest districts of Balochistan province in Pakistan. We published the protocol of the trial prior to commencement [11].

## Methods

### Trial participants

In 2005, we randomly selected 32 enumeration areas (EA) from Lasbela district population census [12]. In each EA, interviewers contacted homes of approximately 100 children under the age of 60 months. The district population is scattered, and each EA comprised four or five villages. After the baseline study [13], a random number generator allocated the baseline communities to 18 intervention and 14 control EAs. The sequence was concealed and the intervention assigned centrally. The intervention group was thus 18 enumeration areas, each of four or five villages and including a total of 3166 children under the age of five years. The 14 control EA, also each of four or five villages, included a total of 2475 children.

### The intervention

In preparation for the intervention the team synthesised the international literature on the likely impact of measles vaccine. Using the baseline study of this trial, it was also possible to estimate the cost and the benefit of recent vaccination in Lasbela district. In the event, measles vaccination in the district proved to have low efficacy [14].

We consulted non-sample communities about how best to present and discuss the evidence from the baseline survey within communities, so that as many households as possible would be drawn into discussing the evidence and perhaps making a positive decision to vaccinate their children as a result. The focus group discussions in these communities indicated clearly that belief in the efficacy of the vaccine was simply not the problem; the overwhelming concern was about the costs of having children vaccinated. Consequently the intervention relied more on the costs of treating measles cases and of having children vaccinated (including travel costs, actual treatment costs but excluding time costs). It cost many times more to treat a child with measles than it did to vaccinate a child against measles (in a ratio of 33:1). Some families, however, paid much less for cases of measles they treated at home (as was the accepted traditional practice in some of the communities). Informed by the discussions in non-sample communities, we developed detailed guides for conducting discussions with community members, to take place in three phases, each phase sometimes requiring several meetings to allow the participants to come to a conclusion from the evidence-based discussion.

We recruited and trained men and women from Lasbela to lead and record the three-phased discussions in the intervention communities. Training for each phase of the intervention lasted two to three days, including classroom sessions and field practice. We trained more people than required for the intervention and selected those who performed best to undertake the work, forming nine field teams, each with male and female members. The field teams completed the discussions in the 18 intervention communities between August 2006 and March 2007. The population of Lasbela is scattered and each community had several villages. The teams organised male and female groups in the 94 villages; a total of 180 community groups, each of 8-10 people, participated in the intervention. The activities of the field teams included: meeting community leaders to explain the purpose of the intervention and seek permission to work in the community; identifying suitable members for the discussion groups; scheduling and facilitating the three phases of discussion (sometimes requiring several meetings for a phase); and assisting the groups to list local barriers to vaccination and develop action plans. The people selected to take part on the discussion groups were trusted within their community and able to convince others about important issues. Generally the same people participated in all the discussion sessions; sometimes additional participants joined after the initial session and a few people were not able to continue through all the sessions.

In the *first phase *the community groups analysed the situation about child vaccination in their union council (the smallest administrative unit within the local government system, and for which we had disaggregated information from the baseline survey). They discussed the prevalence of measles among children and the proportion of children getting vaccinated in their own community, and the importance of childhood vaccinations. The facilitators shared the district level evidence that a child who is not vaccinated has twice the risk of measles, compared with a child who is vaccinated.

The *second phase *discussed evidence on costs and benefits of vaccination from the baseline survey, including the costs of treating a child with measles in comparison with the costs of getting a child vaccinated against measles. The groups also discussed the complications of measles, and benefits and adverse effects of measles vaccination.

In the *third phase *the groups identified the specific challenges and barriers to child vaccination in their own communities and developed plans for actions they could take themselves to address some of these challenges. These included methods for spreading the discussion about vaccination to other community members, as well as ways to increase access to vaccination services, such as sharing transport and helping with childcare. Although the facilitators discussed with participants their plans for disseminating the discussions within their communities, the intervention did not make special provision for the participants to "take back" the discussion to others in the community, relying rather on endogenous networks for the information spill over.

Local supervisors supported and monitored the work of the field teams and documented the outcome of the three phase discussions, using structured checklists and reporting formats. They visited the teams in the field, provided feedback, and assisted them to remedy any problems encountered with the intervention implementation.

During the period of the intervention, the Lasbela government health department implemented a health education programme in the district, aiming to reach all communities, with messages particularly about household hygiene and prevention of diarrhoea in children. Both intervention and control communities received this health education programme, implemented mainly through lady health workers (LHW) and other local officers, who received specific training for this activity.

### Outcomes

The primary outcome was uptake of measles and full DPT vaccination of 12-23 month olds, as reported by the main caregiver. We used well-known local terms for the various vaccinations, and described their timing and administration (for example, "an injection into the upper arm" for measles vaccine) to assist mothers' recall; we did not verify the mothers' reports by checking vaccination cards among those who held these. Secondary outcomes specified per protocol were the theory-based "cascada" of intermediate outcomes leading to vaccination uptake: conscious knowledge, attitudes about vaccination, subjective norms, intention to change, agency/self efficacy, and discussion within the household [11]. In the baseline (spring 2005) and follow-up (spring 2007) surveys, a household questionnaire included questions about vulnerability of the household and questions to mothers concerning their education, childcare knowledge, attitudes and practices.

The field coordinator for the household surveys (MB) knew which clusters had received the intervention but interviewers did not. We did not evaluate the success of this blinding. Only a few people participated in the structured discussion groups but the intention was for these people to widen the discussion, so that most parents in each intervention cluster would know of the structured discussions.

### Analysis

Cluster was the unit of randomisation, intervention and principal analysis. All analyses followed the intention to treat principle, considering all children in designated intervention clusters as exposed irrespective of parental participation in the structured discussions. For the principal analysis of primary outcomes we used an unpaired t-test of vaccination rates children aged 12-23 months in intervention clusters compared with control clusters. We estimated absolute event rates in intervention and control groups, number needed to treat (NNT) and its 95% confidence interval.

An earlier analysis of the baseline survey showed the importance of several factors in the vaccination of children in Lasbela [13]. To adjust for significant baseline differences (see Table 1), we used generalized estimating equation (GEE) in the R package Zelig [15]. Because several factors converged around the distance from facility (including visits from vaccination teams, visits of LHWs and, consequently, information provided by the LHW), we combined distance (less than 5 km) and visit by a LHW in a single variable representing access. Adjusting for the baseline differences - willingness to travel and knowledge of a preventable disease - in an exchangeable correlation structure (logit.gee model, 1000 simulations). Analysis of secondary outcomes followed the same principles.

**Table 1:** Baseline characteristics of intervention and control children aged 12-23 months, with significance tested using a cluster comparison (based on numbers of children in order to assess baseline differences in intervention and control groups).

	Intervention		Control		Cluster analysis (t-test)	Naïve 2 × 2 Mantel Haenszel odds ratio
				
	%	Based on	%	Based on		
Households:						95%CI
Roof type (good)	36%	202/533	28%	125/371	P = 0.451	0.90-1.61
Breadwinner income (good)	50%	263/529	55%	203/367	P = 0.287	0.60-1.05
Low room occupancy	40%	217/532	46%	174/371	p = 0.213	0.59-1.03
Household not vulnerable	36%	195/528	40%	158/367	P = 0.635	0.58-1.03
Head of HH has formal education	35%	198/531	33%	135/371	P = 0.693	0.78-1.39
Vaccination facility within 5 km	55%	308/487	30%	152/364	p = 0.127	1.81-3.22
Village visited by vaccination team	43%	227/491	31%	86/323	P = 0.398	1.74-3.29
Mothers:						
Willing to travel to vaccinate	98%	420/431	89%	278/309	P = 0.009	3.64-98.3
Mother has formal education	8%	48/538	6%	28/373	P = 0.511	0.73-2.11
Have heard about vaccinations	89%	490/537	86%	328/373	P = 0.511	0.90-2.27
Know a vaccine preventable illness	84%	458/534	73%	274/365	P = 0.084	1.41-2.87
Neighbours think vaccination worthwhile	86%	471/537	79%	301/368	P = 0.310	0.96-2.35
Think vaccination worthwhile	94%	512/537	90%	335/366	P = 0.228	0.99-3.51
Ever visited by LHW	32%	198/538	12%	50/373	P = 0.068	2.68-5.56
LHW told about vaccinations	9%	59/533	5%	21/372	P = 0.445	1.26-3.94
Children (12-23 months):						
Measles vaccination	47%	279/537	49%	198/367	P = 0.832	0.70-1.22
DPT-full schedule	51%	304/536	45%	184/366	P = 0.505	0.98-1.71
Polio vaccine in last 12 m	99%	530/537	100%	369/369	P = 0.502	-

We used Amelia II [16] to impute values for missing data with an EM algorithm for all variables included in the GEE model of the primary outcomes. Estimates reconciled data from ten imputed data sets using Rubin's approach [17] in the R package Zelig [18].

### Ethical review

A registered ethical review board in Karachi, Pakistan, approved the study in 2004. A separate ethical review board at the University of Ottawa approved it in 2005.

## Results

The baseline survey contacted 538 children aged 12-23 months in intervention and 373 in control communities. The follow-up survey contacted 536 in intervention and 420 in control communities, the increase in the control communities being because of fuller access to one of the control communities, which was not possible in the baseline survey.

**Figure 1:**
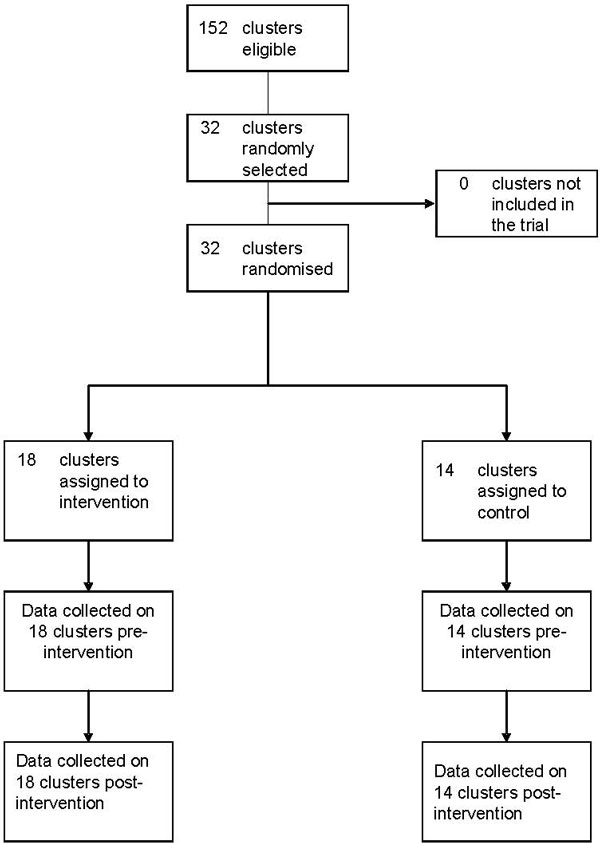
**Consort diagram of clusters and flow through the trial**.

Figure 1 shows the flow of clusters through each stage. There were no deviations from protocol. Table 1 shows the baseline difference between intervention and control groups: knowledge of vaccine protection, visits by LHWs (who visit homes recommending child care activities) and access (less than 5 km from a vaccination post).

Table 2 shows the cluster analysis for measles vaccination (12-23 months of age). Intervention clusters had significantly higher vaccination rates (50% compared with 30%, RD 0.20, 95% CI 0.031-0.372). A similar size difference was evident for full DPT (51.7% compared with 23.2%, RD 0.285, 95%CI 0.141-0.429, Table 3). We detected no meaningful difference in the already high rates of polio vaccination (intervention mean 0.988 based on 524/530, control mean 0.986 based on 415/422; RD 0.002 95%CI -0.0014-0.017).

**Table 2:** Proportion of children (12-23 months) reported to have received measles vaccine.

	Intervention clusters			Control clusters	
1	15/26	0.58	1	26/33	0.79
2	17/24	0.71	2	20/37	0.54
3	25/31	0.81	3	16/35	0.46
4	29/38	0.76	4	11/21	0.52
5	9/26	0.35	5	2/19	0.11
6	34/55	0.62	6	1/28	0.04
7	12/16	0.75	7	10/25	0.40
8	25/44	0.57	8	21/33	0.64
9	29/43	0.67	9	10/37	0.27
10	5/21	0.24	10	0/20	0
11	16/26	0.62	11	1/16	0.062
12	13/19	0.68	12	3/39	0.077
13	0/20	0	13	10/36	0.28
14	16/34	0.47	14	5/41	0.12
15	7/30	0.23			
16	8/36	0.22			
17	16/26	0.44			
18	7/21	0.33			
	283/536	mean 0.50		136/420	0.30

Pooled SD 0.234, Difference 0.20, 95% 0.031-0.372 t = 2.413, 30 df p = 0.0221. NNT = 5, 95%CI 3-31.

**Table 3:** Proportion of children (12-23 months) reported to have received full course of DPT (diphtheria, pertussis, tetanus) vaccine.

	Intervention clusters			Control clusters	
1	14/26	0.54	1	19/33	0.58
2	20/24	0.83	2	15/37	0.41
3	17/30	0.56	3	14/35	0.4
4	27/38	0.71	4	10/22	0.45
5	6/26	0.23	5	1/19	0.05
6	29/55	0.53	6	1/28	0.04
7	9/16	0.56	7	9/25	0.36
8	24/44	0.54	8	6/33	0.18
9	30/43	0.70	9	10/36	0.28
10	6/21	0.29	10	0/20	0
11	17/26	0.65	11	1/18	0.056
12	15/19	0.79	12	4/39	0.10
13	1/20	0.05	13	8/36	0.22
14	19/34	0.56	14	5/42	0.12
15	13/30	0.43			
16	11/37	0.30			
17	18/26	0.69			
18	7/20	0.35			
	283/535	0.517		103/422	0.232

Difference 0.285 95%CI 0.141-0.429 t = 4.06 30 df p = 0.0003. NNT3.5, 95%CI 2-7.

Adjusting for baseline differences using a logit model generalised estimating equation, the intervention effect remained high (first difference 0.19, 95% CI 0.05-0.32). This represents doubling of the odds of measles vaccination in the intervention communities (OR 2.20, 95% CI 1.24-3.88). Adjusting the effect on DPT3 vaccination by the baseline differences, the first difference dropped slightly from 28.5% to 27.0% (95% CI 0.162-0.38). This corresponds to three-fold odds of completing DPT vaccination among the intervention group compared with the controls (OR 3.36, 95% CI 2.03-5.56), after adjusting for baseline differences.

To test the effect of missing data on the primary outcomes, we remodelled the GEE estimates across 10 data sets with missing values imputed from other variables. The impact of measles vaccination remained constant (RD 0.1864, 95% 0.0417 - 0.3311), as did that for full DPT vaccination (RD 0.2674, 95% 0.1449 - 0.3746). The initial models included willingness to travel, knowledge of a vaccine preventable illness and access (a composite variable combining distance less than 5 km and visit by a LHW). The final models for both included only the intervention and access.

**Figure 2:**
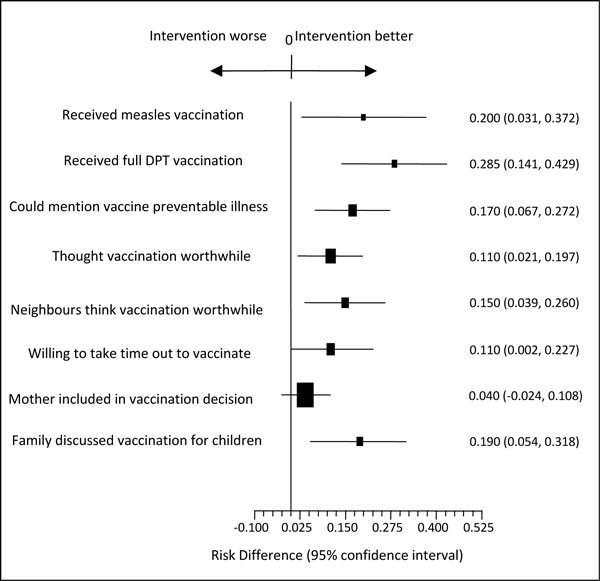
**Secondary impacts of the intervention**.

The analysis of secondary outcomes, per protocol, dealt with each of a "cascada" of precursors to vaccination uptake [11,19]. Table 4 shows a significant impact on conscious knowledge and attitudes about vaccination, subjective norms, intention to change, and discussion in the home. These results are summarised in Figure 2. The GEE analysis of secondary outcomes, adjusting for the same baseline differences, confirmed these findings.

**Table 4:** Cluster analysis of secondary outcomes (cascada) among parents of children aged 9-60 months (t-test of difference between 18 intervention and 14 control sites, cluster analysis as in Tables 2 and 3).

	Intervention clusters	Control clusters	Outcome in primary (cluster) analysis (t-test)	GEE adjusting for baseline differences
Respondent could mention an illness preventable by mean 0.74	2368/3153 mean 0.74	1437/2431 mean 0.58	Difference 0.17, 95% 0.067-0.272 t = 3.369, 30 df p = 0.002	Difference 0.121 95% 0.055-0.189 (1)
Do you think it's worthwhile to vaccinate children? (attitude)	3006/3161 mean 0.95	2116/2475 mean 0.84	Difference 0.11, 95% 0.021-0.197 t = 2.543, 30 df p = 0.016	Difference 0.054 95% 0.013-0.105 (1)
Do your neighbours think it's worthwhile to vaccinate children? (subjective norm)	2842/3166 mean 0.89	1884/2475 mean 0.74	Difference 0.15, 95% 0.039-0.260 t = 2.755, 30 df p = 0.010	Difference 0.095 95% 0.011-0.182 (1)
How much time are you prepared to spend to take a child from your household to be vaccinated? Willing to take some time (intention)	2954/3088 mean 0.95	2037/2317 mean 0.84	Difference 0.11, 95% 0.002-0.227 t = 2.086, 30 df p = 0.046	Difference 0.073 95% 0.015-0.156 (2)
Mother included in decisions about vaccination (agency)	1834/3131 mean 0.59	1345/2434 mean 0.54	Difference 0.04, 95% -0.024-0.108 t = 1.299, 30 df p = 0.204	Difference 0.043 95% -0.009-0.097 (NS) (3)
Have you discussed vaccination for children in the family? (discussion)	1584/3155 mean 0.49	826/2459 mean 0.31	Difference 0.19, 95% 0.054-0.318 t = 2.868, 30 df p = 0.007	Difference 0.155 95% 0.032-0.270 (2)

Initial model: intervention, willingness to travel, access, knowledge of preventable disease.

(1) Final model: intervention, willingness to travel, access.

(2) Final model: intervention, knowledge of preventable disease.

(3) Final model: intervention, knowledge of preventable disease, access.

## Discussion

The results support the hypothesis that evidence-based structured community discussions can increase vaccine uptake without relying on improvements of health service delivery. In a context of falling vaccination coverage, the intervention maintained rates at the baseline level. Compared with control communities, this doubled the odds of 12-23 month old children receiving measles vaccination and tripled the odds of completing DPT vaccination. It will be important to examine the impact of the intervention where vaccination uptake is not falling.

We adjusted the findings of the conservative cluster analysis by baseline differences. We did not control for covariants in the follow-up survey, as these could constitute part of the causal chain. For example, adjusting for whether households "had discussed vaccination in the household" would reduce the measured intervention effect; under the hypothesis, vaccination uptake is a consequence of discussions in the household, which is, in turn, a consequence of the structured community discussions.

In the Lasbela context, where very few families have up to date health records, we had to rely on the caregiver's report of vaccination uptake. Authors from developed countries have argued that maternal recall is inadequate compared with health facility records [20,21]. Studies from developing countries contrast this, concluding that reliance on mothers' reports gave accurate estimates of coverage in Egypt [22], Sudan [23], Guatemala [24] and Costa Rica [25]. A study in India found that maternal recall underestimated children's vaccination status but using vaccination cards was not helpful because less than half the mothers had cards and the cards were often incomplete or grossly inaccurate [26].

Even if maternal recall is adequate for estimating coverage, it is theoretically possible that those exposed to the intervention overstated uptake - a halo effect. However, we consider this unlikely. First, the intervention only directly involved a few participants, and the further spread of discussion came from within the community. At about the same time, both intervention and control communities received visits promoting child and household hygiene. Second, the communities continue to request that the mobile polio vaccination teams should also offer measles vaccine; overstating uptake does not fit with this. Both intervention and control groups already had very positive views of vaccination in the baseline studies (Table 1).

Knowledge translation has increased reported vaccination uptake in other settings, although often with some accompanying changes to service delivery. In Ghana, home visits to engage people in discussions about vaccination increased uptake in towns with relatively low coverage rates [27]. A similar door-to-door approach claimed a positive impact in Mozambique [28]. "Village-resource rooms" were successful in improving knowledge in the West Bank, although they did not increase vaccination uptake [29]. In the Philippines, a mass media campaign claimed to increase vaccine uptake by 11% [30]. And in Bangladesh, advocacy among women by a credit programme increased measles vaccine uptake by 9% [31].

We viewed the secondary outcomes as precursors of vaccination uptake. The convincing impact of the intervention on these offers useful supportive evidence for a causal linkage between the intervention and vaccine uptake. The single exception was the variable used to measure self-efficacy or agency to take up vaccination, inclusion of the mother in decisions about childhood vaccination. This could reflect a local lack of influence of women in decisions relating to the health of their children; or it could reflect the weakness of our indicator of agency.

Apart from this, the fact that the intervention significantly changed all steps in the *cascada *(Figure 2) is compatible with the intervention changing behaviour in a reasoned way: conscious knowledge increased, attitudes towards vaccination improved, subjective norms improved as did intention to vaccinate and discussion of the value of vaccination.

The structured discussion rounds sometimes led to action plans in the intervention communities beyond stimulating discussion about vaccinations within households. Particularly in those villages with poor access to vaccination services, plans included sharing transport to vaccination points and providing care for some children while parents took others to be vaccinated. These community initiatives may have helped to maintain vaccination levels in the face of generally falling levels.

We estimated the direct costs of implementation of the intervention within Lasbela, with six field teams undertaking the three phased discussions in 18 communities (94 villages), with a total of 180 community groups. Including direct field supervision but excluding the costs of provincial and national coordinators working on the project, the intervention cost US$63,600. This does not include the costs of the baseline and follow up surveys. Based on our experience with supporting a district government health education programme in the district, the district government could implement the knowledge translation intervention throughout Lasbela district - where there are around 10,000 children in the 12-23 month age group - for the equivalent of US$90,000 ($9 per child vaccinated in the target age group).

## Conclusion

New vaccines, the investment emphasis of the global vaccine initiative, are unlikely to reach children not already receiving existing vaccines. Implementation research is urgently needed to inform strategies to increase vaccine uptake, especially in those parts of the world where vaccination coverage is low or even decreasing. We would not expect the exact intervention applied here, based on specific results of the baseline study, to be applicable elsewhere, but the approach might be so.

We involved only a few participants directly in the evidence-based discussions about costs and benefits but, not a trivial finding, the impact was measurable beyond that. It is possible that involving greater numbers of people in structured discussions directly in each community could increase the vaccination uptake further. Action plans developed in some communities suggest the intervention may also improve the terms of engagement between communities and service providers.

The household cost-benefit equation is a lens for understanding and negotiating parental decisions about vaccination: people weigh things up before making their health choices. This household equation for childhood vaccination can also be modified by appropriate knowledge translation without relying on improved services. In the Lasbela case, the pre-intervention household cost-benefit equation might well have taken into account the low efficacy, and the household opted not to invest in it. The trial set out to show an increase in the *demand side *of uptake, and we believe we achieved that. The remaining uncomfortable truth is that even if this is possible, it is often still important to increase the efficacy through improved service delivery quality. Future research should focus on both demand and supply side interventions, alone and in combination.

## List of abbreviations used

EA: Enumeration area; NNT: Number needed to treat; GEE: Generalized estimating equation; LHW: Lady health worker.

## Competing interests

The authors declare that they have no competing interests.

## Authors' contributions

NA designed the study, undertook the analysis and drafted the report. AC assisted with the design and analysis, supported the surveys and intervention, and helped to draft the report. KO was responsible for the surveys and for data management, and reviewed the report. NMA helped manage the surveys, led the intervention and reviewed the report. MB coordinated the field teams for the surveys and intervention and reviewed the report. AHF reviewed the design, assisted with the analysis and reviewed the report. BS reviewed the design, advised on the analysis and reporting. GAW reviewed the design, advised on the analysis and reporting. JLS reviewed the design, advised on the analysis and reporting.
